# Assessment of the submandibular fossa depth and diameter of the mandibular canal via cone beam computed tomography: a comparative study

**DOI:** 10.1186/s40902-025-00473-w

**Published:** 2025-08-12

**Authors:** Shilpa Levingston, Shivaprasad, Devika Shetty, Aneesa Ayoob, Shruthi M

**Affiliations:** 1 Department of Oral Medicine & Radiology, Srinivas Institute of Dental Sciences, Mukka, India; 2 Rajiv Gandhi University of Health Sciences, Bengaluru, India; 3 Department of Periodontology, Manipal College of Dental Sciences, Manipal Academy of Higher Education, Manipal, India; 4 Amrita Vishwa Vidyapeetham University, Coimbatore, India

**Keywords:** Cone-beam computed tomography, Mandible, Radiology

## Abstract

**Introduction:**

The submandibular fossa (SF), a depression on the lingual surface of the mandible extending from the mental foramen to the molar region, accommodates the submandibular salivary gland, influencing its depth and shape. Accurate knowledge of this region is essential for reducing complications during oral surgeries, such as implant placement and extractions. This study was aimed to assess SF depth, mandibular canal (MC) diameter, and concavity angles between males and females via cone-beam computed tomography (CBCT).

**Methodology:**

CBCT scans of 160 patients (80 males and 80 females) aged 18–35 years were analysed. SF depth was classified into three types: Type I (< 2 mm), Type II (2–3 mm), and Type III (> 3 mm). The MC diameter and concavity angles were measured in the interradicular region of the mandibular molars. The data were statistically analysed via unpaired t tests and chi-square tests (*p* < 0.05 was considered significant).

**Results:**

Males presented greater mean SF depth, MC diameter, and concavity angles than females did. SF depth was generally more pronounced on the left side in both sexes. Type I SF was the most frequently observed SF depth classification.

**Conclusion:**

CBCT provides valuable insights into mandibular anatomy. Although certain anatomical differences were observed between sexes, particularly in MC diameter, not all findings reached statistical significance. These results suggest the importance of individualized radiographic assessment during surgical planning.

## Introduction

The submandibular fossa (SF), which results from the pressure exerted by the submandibular salivary gland against the lingual cortex of the mandibular body, is a frequently observed anatomical variation in the mandible. This concavity typically arises from the distal region of the inferior alveolar foramen, is positioned inferior to the mylohyoid line, and extends posteriorly towards the region of the mandibular third molars [1]. The submandibular gland occupies this anatomical depression in the mandible. Different imaging modalities, such as panoramic imaging, digital volume tomography (DVT), and conventional computed tomography (CT), are routinely used to evaluate the morphology and dimensions of this region [2].

The posterior mandibular region, including the inferior dental nerve and the submaxillary fovea in the posterior mandibular region, forms a critical anatomical zone by virtue of the presence of vital vascular structures, namely, the submental and sublingual arteries. Any injury to the inferior alveolar canal, particularly during implant placement, can result in neurosensory disturbances such as paraesthesia, manifesting as numbness of the chin and the commissure of the mouth [3]. Additionally, disruption of the neurovascular bundle may compromise the vitality of multiple teeth within the affected quadrant. Furthermore, perforation of the lingual cortical plate during surgical interventions involving the submandibular fossa may precipitate significant haemorrhage and subsequent hematoma formation, potentially leading to life-threatening airway obstruction and other complications [4–7].

Traditionally, to assess the anatomical areas of various regions, osteometry, diagnostic casts, and palpation of ridges are performed, and several imaging techniques are available for assessing bone volume and quality during dental implant planning. One such modality is panoramic radiography, which offers a broad overview of the jaws and is often the first-choice imaging modality due to its widespread availability, affordability, and efficiency in preliminary evaluations. However, panoramic images have notable limitations, such as their inability to accurately depict the buccolingual contour of the ridge, diminished visibility of cortical bone plates and concavities, and inconsistent image magnification [8].

Therefore, CBCT plays an important role in understanding the status of concavities and selecting a proper fixture. Care must be taken during implant surgeries to properly evaluate drill angulation and implant positioning via radiographic imaging. For more precise presurgical planning, computed tomography and, more recently, CBCT have been preferred. Compared with traditional CT, CBCT produces detailed cross-sectional images with a lower radiation dose and lower cost. This technology enables clinicians to accurately evaluate the buccolingual dimensions of the implant site through multiplanar reconstructions.

Previous studies have individually explored these anatomical structures, such as visualizing the mandibular canal and identifying the submandibular fossa [9–12]. But limited research has explored the interplay between submandibular fossa depth, mandibular canal diameter, and concavity angle from a gender perspective within a younger adult population. The young adult bone morphology is relatively stable and free from the degenerative changes typically seen in older adults, thus allowing a clearer anatomical baseline. Understanding gender-based anatomical differences at this stage provides reference data that may be valuable for preventive and early intervention strategies, orthodontic planning, or future implant considerations. Moreover, this demographic is increasingly seeking elective implant procedures due to trauma, congenital absence, or early loss of teeth unrelated to periodontitis. Therefore, this study aimed to evaluate and compare submandibular fossa depth, mandibular canal diameter, and the concavity angle between males and females via CBCT.

## Methodology

### Study design, study setting and study participants

This was a retrospective cross-sectional study conducted on CBCT scans of 160 patients (80 males and 80 females), aged 18–35 years, who were referred for mandibular imaging between November 2018 and July 2020. CBCT scans were selected retrospectively from patients who had undergone imaging for diagnostic purposes unrelated to this study, such as implant planning or third molar evaluation. Prior ethical clearance was obtained from the institutional ethical committee. Informed consent was obtained from the included participants. A convenience sampling method was adopted to select individuals for this study.

### Eligibility criteria

The study included patients aged 18 to 35 years with mandibular posterior teeth and fully erupted permanent teeth with complete root formation. Subjects were excluded if they had missing mandibular posterior teeth, radiographs showing mandibular bone pathologies or fractures, periodontitis, periapical pathologies, a history of maxillofacial trauma, orthognathic surgery, reconstructive surgery in the region of interest, developmental defects in the area, or CBCT images with distortion or artefacts. Patients with periodontitis were excluded to avoid confounding effects of inflammatory bone loss on mandibular anatomy. This ensured more accurate and consistent baseline measurements of the submandibular fossa and mandibular canal.

### Measurements of CBCT imaging

For CBCT imaging, the NewTom VGi evo Machine was operated at 110 kVp and 5.3 mA for 18 s. The images were displayed on a personal computer equipped with a 27-inch LED monitor and integrated NNT viewer software. The measurements were conducted via NNT viewer software for CBCT. All the CBCT images were evaluated independently by two trained oral radiologists. To assess intra observer reliability, 20% of the images were re-evaluated after a 2-week interval. Inter and intra-observer agreement was measured via the intraclass correlation coefficient (ICC), with values > 0.80 considered acceptable.

**Fig. 1:**
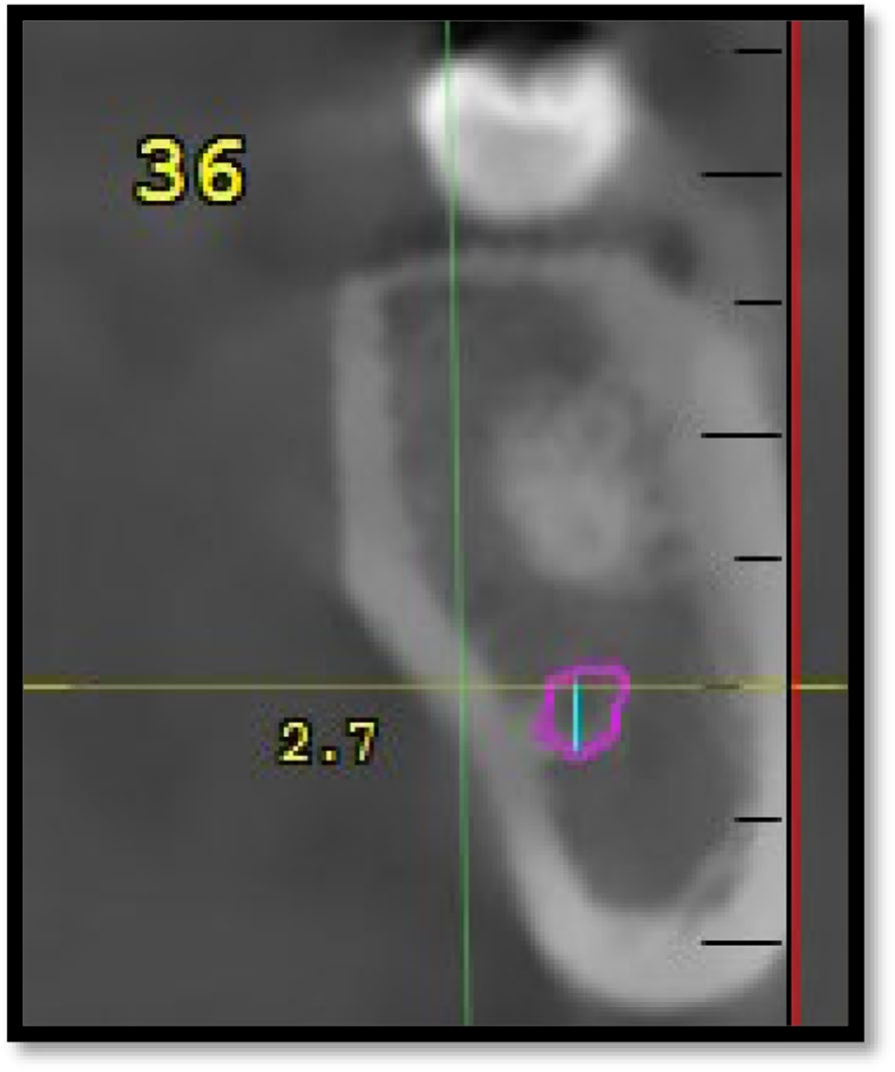
Diameter of mandibular canal

Using NNT software for CBCT, 160 CBCT images (320 hemimandibles) were evaluated in cross-sections in relation to the mandibular 1 st molar and the 2nd molar teeth in the interradicular region. CBCT images were evaluated in standardized coronal cross-sections at the interradicular regions of the first and second mandibular molars on both sides (Fig. 1).

The right and left mandibular sides were analysed separately to account for potential anatomical asymmetries, which may be developmental, functional, or habitual in origin. Asymmetry in mandibular bone morphology has been reported in the literature and can influence surgical risk, particularly in procedures such as implant placement where precise anatomical mapping is critical. Evaluating both sides independently also enhances diagnostic accuracy in site-specific treatment planning.

**Fig. 2:**
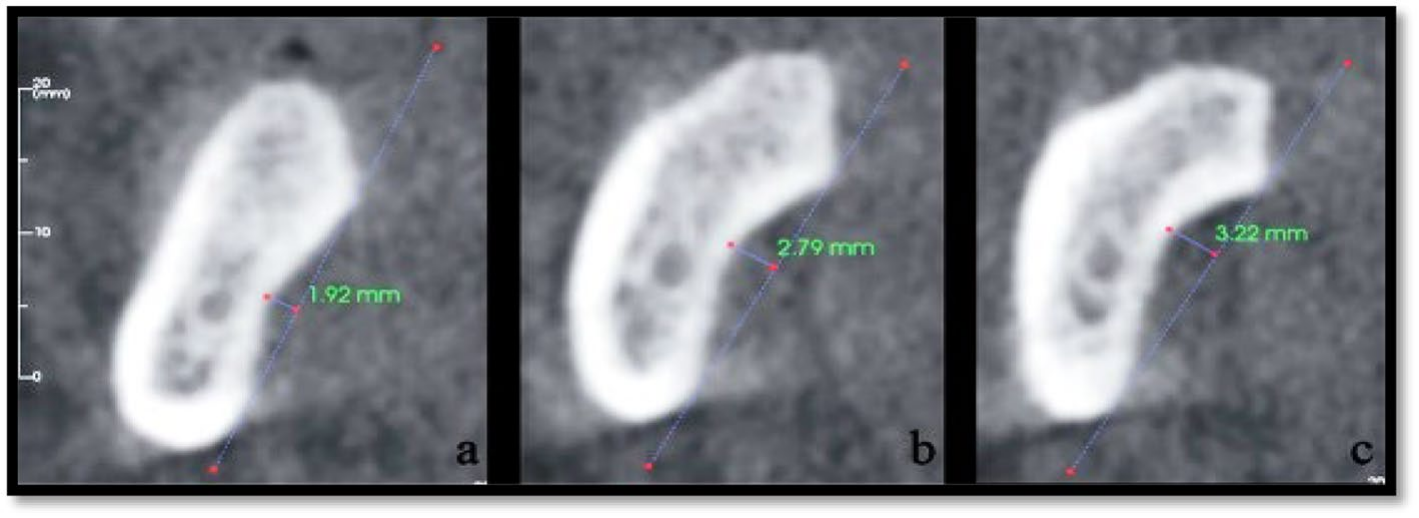
Types of submandibular fossa (**a** type I, **b** type II, **c** type III)

Submandibular fossa (SF) depth was measured from the deepest point of the lingual concavity to a line connecting the most prominent superior and inferior borders of the lingual cortex via coronal sections. A line “A” is placed on the most prominent superior and inferior points of the lingual concavity, and a second line “B” is drawn from the deepest point of the concavity perpendicular to the first line. The SF types are then classified as follows: Type I, < 2 mm; Type II, 2 to 3 mm; and Type III, > 3 mm (Fig. 2).

**Fig. 3:**
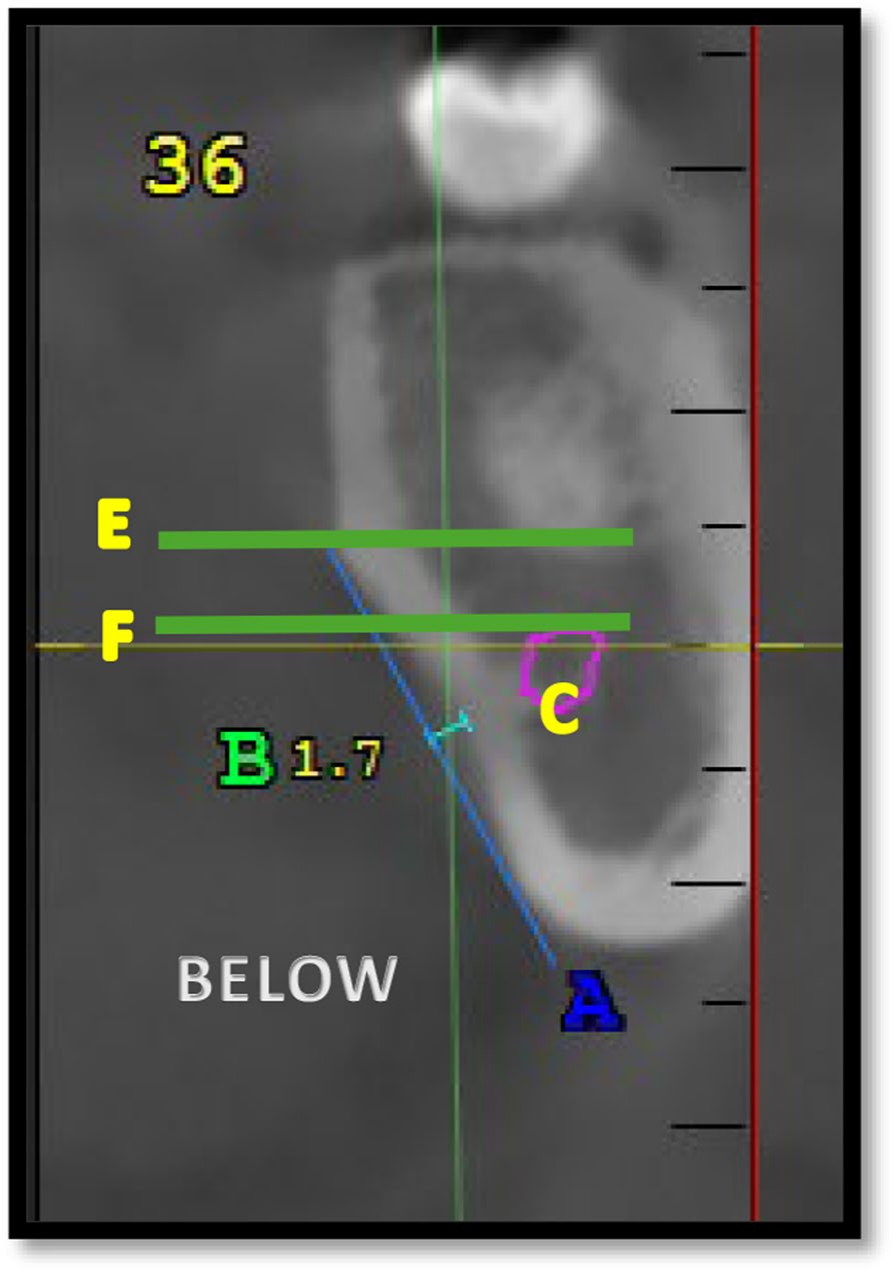
Position of the deepest region of submandibular fossa compared to mandibular canal

The position of the deepest region of the submandibular fossa compared with the mandibular canal is determined by placing 2 lines, “E” and “F”, tangential to the upper and lower walls of the mandibular canal and parallel to the horizon in relation to the 1 st molar teeth in the interradicular region; these lines are then evaluated and categorized into 3 groups: whether the deepest region lies above the canal (above E), the same level as the canal (between E and F), and below the canal (below F) (Fig. 3).

**Fig. 4:**
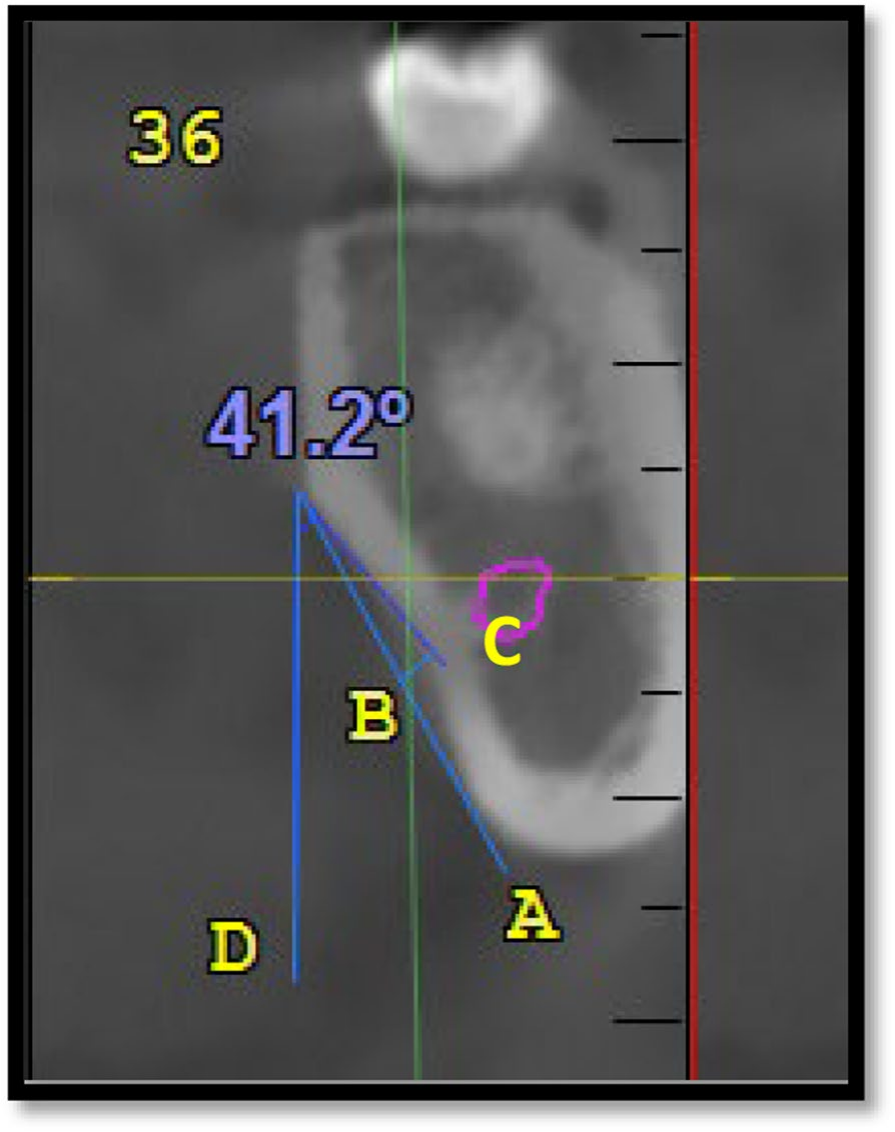
Concavity angle

To measure the concavity angle, a line was drawn tangential to the upper point of the concavity and perpendicular to the horizon (Line C). A line is subsequently drawn tangential to the upper wall of the concavity connecting the upper and lower points of the submandibular gland fossa (Line D); the angle between lines C and D is measured in relation to the 1 st and 2nd molar teeth in the interradicular region (Fig. 4).

### Sample size estimation

**Table:** 

**Analysis:**	A priori: Compute the required sample size
**Input:**	Tail(s) = Two
	Effect size d = 0.4566408
	α err prob = 0.05
	Power (1-β err prob) = 0.80
	Allocation ratio N2/N1 = 1
**Output:**	Noncentrality parameter δ = 2.833382
	Critical t = 1.975694
	Df = 152
	**Sample size group 1 = 77**
	**Sample size group 2 = 77**
	**Total sample size = 154**
	Actual power = 0.803856

A power analysis was performed with G*power, version 3.0.1 (Franz Faul universitat, Kiel, Germany).

### Statistical analysis

The data were analysed via IBM SPSS Statistics version 22. The Shapiro‒Wilk test was performed to assess normality. For normally distributed data, independent t tests were used for comparisons between males and females. Nonparametric data were analysed via the Mann–Whitney U test. The chi-square test was applied to compare categorical variables such as SF depth types. A *p* value of < 0.05 was considered to indicate statistical significance.

## Results

A total of 160 patients, with an average age of 18–35 years, were included in this study, with an equal distribution of male and female patients (80 males and 80 females). A comparative radiographic study was conducted on male and female patients on the left and right sides of the posterior mandibular region via CBCT images. The submandibular fossa depth, concavity angle and diameter of the mandibular canal in the 1 st molar and 2nd molar regions and the deepest region of the submandibular fossa were compared with those of the mandibular canal in the 1 st molar region.

**Fig. 5:**
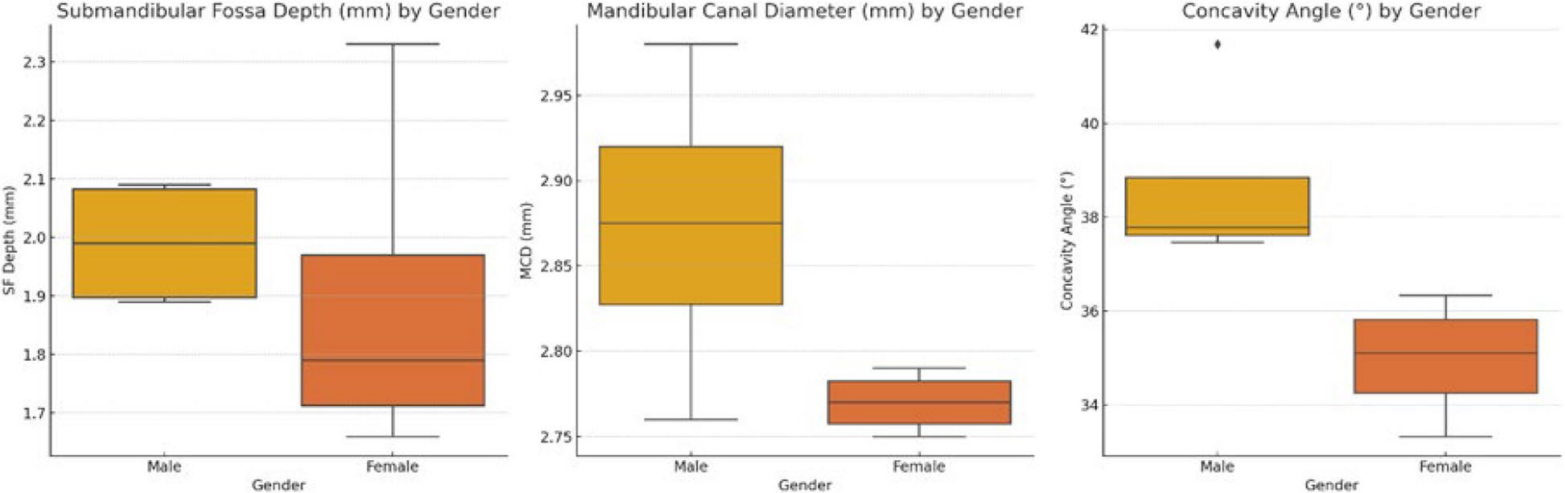
Distribution of submandibular fossa (SMF) depth types and deepest SMF locations by sex

The data presented in Table 1 highlight the differences in the depth types of the submandibular fossa (SMF) at the mandibular 1 st and 2nd molar intra radicular regions, as well as the location of the deepest region of the SMF relative to the mandibular canal, by sex. For the mandibular 1 st molar intra radicular region, both the right and left sides show that Type I depth is more common in females (75% and 68.8%, respectively) than in males (62.5% and 61.3%). However, these differences are not statistically significant. The Type II depth has a similar distribution between the sexes, whereas the Type III depth is rare and is found predominantly in males. In contrast, significant sex differences were observed in the mandibular 2nd molar interradicular region. On the right side, Type I depth is significantly more prevalent in females (66.2%) than in males (35%), whereas Type II depth is more common in males (55%) than in females (33.8%). The left side shows a similar trend, with females having a greater prevalence of Type I depth (65%) than males do (42.5%) and males more commonly exhibiting Type II depth (56.2%) than females do (35%). Type III depth is rare and is predominantly found in males. With respect to the location of the deepest region of the SMF at the mandibular 1 st molar, females are more likely to have the deepest region located above the mandibular canal on both the right (67.5%) and left (75%) sides than males are (48.8% and 61.3%, respectively), although these differences are not statistically significant (Fig. 5).

**Table 1:** Distribution of submandibular fossa (SMF) depth types and deepest SMF locations by sex (*n* = 160)

Region/Parameter	Type/Location	Males n (%)	Females n (%)	Chi-square	*P* value
SMF Depth – 1 st Molar (Right)	Type I	50 (62.5%)	60 (75.0%)	5.06	0.080
	Type II	24 (30.0%)	19 (22.8%)		
	Type III	6 (7.5%)	1 (1.2%)		
SMF Depth – 1 st Molar (Left)	Type I	49 (61.3%)	55 (68.8%)	5.36	0.068
	Type II	26 (32.5%)	25 (31.2%)		
	Type III	5 (6.2%)	0 (0.0%)		
SMF Depth – 2nd Molar (Right)	Type I	28 (35.0%)	53 (66.2%)	19.78	0.000
	Type II	44 (55.0%)	27 (33.8%)		
	Type III	8 (10.0%)	0 (0.0%)		
SMF Depth – 2nd Molar (Left)	Type I	34 (42.5%)	52 (65.0%)	8.72	0.013
	Type II	45 (56.2%)	28 (35.0%)		
	Type III	1 (1.2%)	0 (0.0%)		
Deepest SMF – 1 st Molar (Right)	Above	39 (48.8%)	54 (67.5%)	5.90	0.052
	At level	36 (45.0%)	22 (27.5%)		
	Below	5 (6.2%)	4 (5.0%)		
Deepest SMF – 1 st Molar (Left)	Above	49 (61.3%)	60 (75.0%)	4.44	0.109
	At level	29 (36.2%)	17 (21.2%)		
	Below	2 (2.5%)	3 (3.8%)		

*SMF* Submandibular Fossa

Grouped bar chart comparing the frequency (%) of SMF depth types (types I, II, III) in the mandibular 1 st and 2nd molar regions and the relative location of the deepest point of the SMF (above, at level with, or below the mandibular canal) between males and females. Females more frequently exhibited Type I depths and SMF locations above the canal, particularly in the 2nd molar region, whereas males presented a greater prevalence of deeper concavities (Types II and III).

The rationale for separate evaluation of the right and left sides of the mandible stems from the potential for natural anatomical asymmetry. Although CBCT imaging is site-specific in clinical implant planning, this comparative analysis aimed to identify broader patterns or deviations in morphology that may be relevant for bilateral surgical planning or population-based reference. While some side-wise differences in SF depth and mandibular canal diameter were observed, they were not universally statistically significant, highlighting the need for individualized assessment despite general trends.

**Fig. 6:**
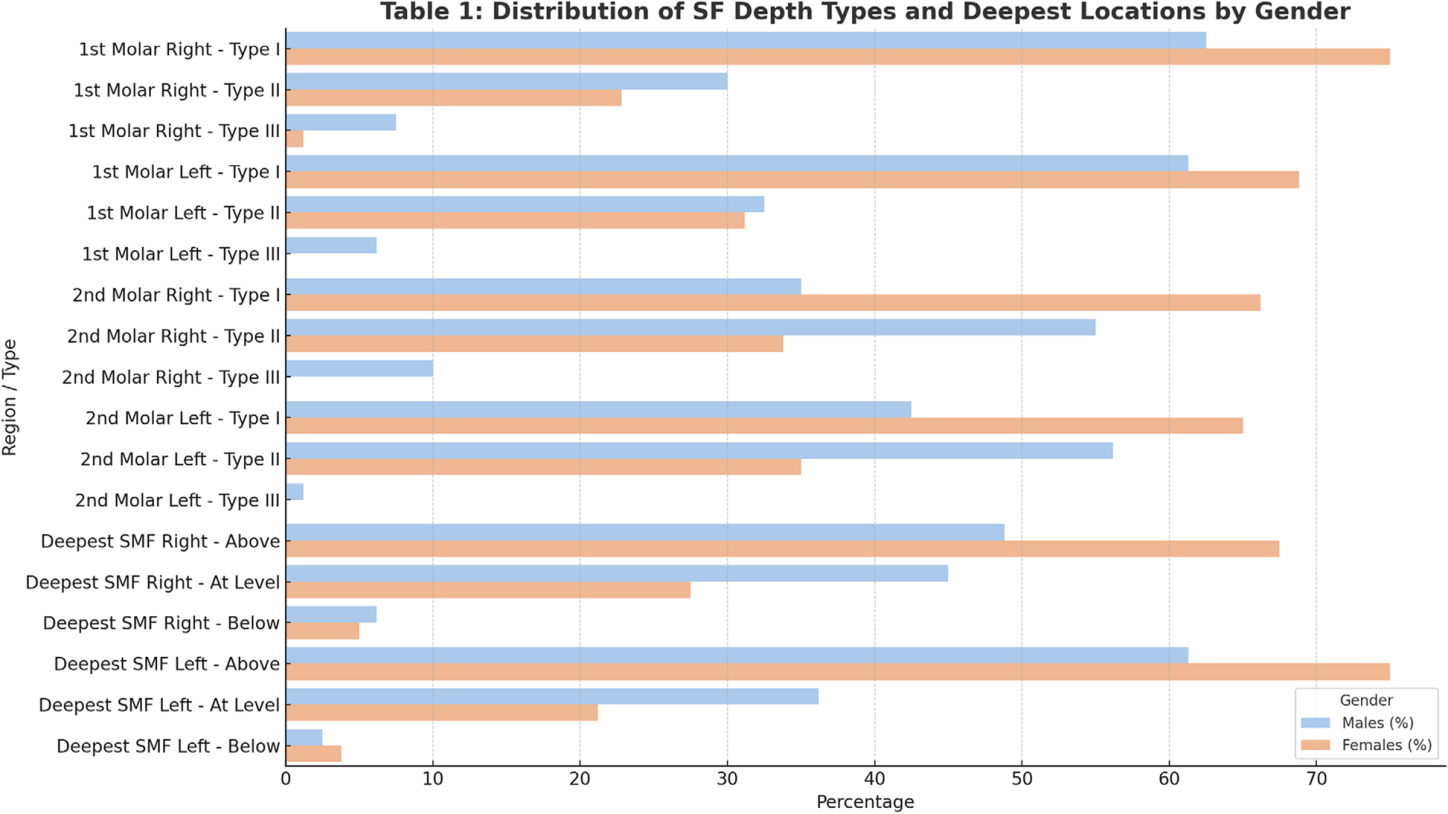
Comparative graphical representation of submandibular fossa depth, cavity angle, and mandibular canal diameter across sex and side. This graph visually summarizes the mean measurements across the right and left mandibular regions in males and females, highlighting significant sex differences, particularly in mandibular canal diameter on the right side

The data presented in Table 2 provide a comparative analysis of the submandibular fossa (SMF) depth, concavity angle, and diameter of the mandibular canal between males and females on both sides of the mandible. For the 1 st molar region, the results revealed no significant differences in the SMF depth or concavity angle between genders on either side. However, the diameter of the mandibular canal on the right side was significantly greater in males (mean = 2.98 mm) than in females (mean = 2.78 mm), with a *p* value of 0.003, indicating a notable sex difference in this specific measurement. In the 2nd molar region, the findings indicate no significant differences between males and females in terms of SMF depth, concavity angles, or mandibular canal diameter (Fig. 6).

**Table 2:** Comparison of submandibular fossa depth, concavity angle, and mandibular canal diameter between males and females

Tooth Region	Variable	Gender	Side	Mean	SD	Mean Diff	*P* value
1 st Molar	SF Depth (mm)	Male	Right	1.89	0.77	−0.12	0.88
		Male	Left	1.90	0.51		
		Female	Right	1.66	0.52	−0.06	0.41
		Female	Left	1.73	0.47		
	Concavity Angle (°)	Male	Right	37.46	10.86	−4.21	0.17
		Male	Left	41.68	28.41		
		Female	Right	33.33	8.81	−1.23	0.16
		Female	Left	34.56	9.63		
	MC Diameter (mm)	Male	Right	2.98	0.47	+ 0.21	0.003
		Male	Left	2.76	0.50		
		Female	Right	2.78	0.44	−0.01	0.87
		Female	Left	2.79	0.52		
2nd Molar	SF Depth (mm)	Male	Right	2.08	0.59	−0.01	0.87
		Male	Left	2.09	0.59		
		Female	Right	1.85	0.45	−0.47	0.36
		Female	Left	2.33	4.64		
	Concavity Angle (°)	Male	Right	37.90	11.28	+ 0.23	0.86
		Male	Left	37.66	9.91		
		Female	Right	36.33	8.84	+ 0.67	0.41
		Female	Left	35.65	8.78		
	MC Diameter (mm)	Male	Right	2.90	0.62	+ 0.05	0.55
		Male	Left	2.85	0.59		
		Female	Right	2.75	0.43	−0.01	0.87
		Female	Left	2.76	0.53		

*SF* Submandibular fossa, *SD* Standard deviation, *MC* Mandibular canal

## Discussion

In this study, the most common SMF depth type in males and females in the mandibular 1 st molar region was Type I, followed by Type II and Type III. This findings is similar to those reported by Bayrak et al. (2017), who also concluded that Type I as the most prevalent depth type, followed by Types II and III, in a study of 500 patients [6]. Another study by Parnia F et al. reported contrasting results, where the deeper SMF depths (Type II and Type III) were more common, occurring in 80% of their study population [13]. These differences may be attributed to variations in the study populations and the methodology implemented. In the current study population, the mandibular 2nd molar region showed significant sex differences, where females exhibited a predominance of Type I depth, whereas males presented greater Type II depth. Type III depths are rare and are found principally in males. These findings contrast with those of a study from Rajput BS et al., which reported that the most common type was Type II, which was followed by Type I and Type III, with Type II exhibiting a notably higher percentage [14]. The dissimilarities observed between these studies could be due to disparities in demographic factors, such as age, racial background, and the specific regions of the mandible examined. This study revealed that SMF depth is generally greater in males than in females in the 1 st and 2nd molar regions. Interestingly, compared with their male counterparts, females presented greater SMF depth in the 2nd molar area on the left side.

Additionally, in both males and females, the SMF depth was greater on the left side for the 1 st and 2nd molar regions, which corresponds with the findings of the study by Ramaswamy et al., who reported that the SMF depth was more pronounced on the left side of the mandible [3]. However, Yildiz et al. (2014) reported that the SMF depth was greater on the right side, highlighting the variability in the SMF depth across different study populations [15]. The present study also revealed that males had a notably larger mandibular canal diameter on the right side in the 1 st molar region than females did, which was in line with the findings of Juodzbalys et al., who reported that sex, age, and race influence mandibular canal variations [16]. A similar study by Ogawa H. et al. noted that the size and location of the mandibular canal are affected by age and sex [17].

A statistically significant finding in this study was the mandibular canal diameter, which was greater on the left side than on the right side in the 1 st molar region of males. These differences were not statistically significant for the mandibular canal diameter, which was greater on the left side in both the 1 st and 2nd molar regions. However, in females, the mandibular canal diameter was greater on the left side in both the 1 st and 2nd molar regions, although these differences were not statistically significant. These findings are consistent with the results of Panjnoush et al., who reported that males had larger concavity angles, and Sina Haghanifar et al. reported that the concavity angle was slightly greater on the right side in female patients [18, 19]. These These differences may be due to variations in measurement methods, racial differences, and study populations in the mandibular 1 st molar region.

In the right and left sides of the mandibular 1 st molar region, the deepest region of the SMF relative to the mandibular canal was more frequently located above the canal in females than in males, although these differences were not statistically significant. This finding is similar to the results of Sina Haghanifar et al., who reported that the deepest regions of the SMF were above the level of the mandibular canal in both sexes [19]. However, Ramaswamy reported that in females, the deepest regions were most commonly at the level of the mandibular canal, followed by below and then above the level of the canal, whereas in males, the deepest regions were most commonly below the level of the mandibular canal [3].

The SMF and concavity angles in this study depict major sex differences and mandibular canal diameter variations, especially on the right side of the 1 st molar region, which is of prime clinical importance. During dental surgical procedures involving this region, variations in the location of the submaxillary fovea should be considered to avoid complications and enhance treatment outcomes. The importance of demographic factors and measurement methodologies in anatomical research is highlighted in this study by highlighting the differences observed.

While CBCT imaging is a routine component of pre-implant planning, this study contributes novel insights by documenting statistically analysed, gender-specific morphometric variations in a demographically uniform, younger adult population. Previous studies have generally focused on older populations with variable bone loss and remodelling patterns, potentially confounding anatomical interpretations. By isolating a healthy, dentate, and skeletally mature cohort, this study provides foundational anatomical data that could serve as a comparative reference for future studies assessing age-related or pathological changes in mandibular morphology.

A limitation of the present study is the exclusion of patients with periodontitis, which may affect the generalizability of the findings. However, this criterion was necessary to ensure anatomical consistency and avoid measurement distortions caused by active or past bone loss. Future studies could compare morphometric data between periodontally healthy and diseased individuals to assess the extent of such variations.

## Conclusion

This study identified anatomical variations in the submandibular region between males and females, with males generally exhibiting greater SF depth, MC diameter, and concavity angles. The Type I SF depth was the most common depth across the sample. While some measurements, such as the MC diameter, showed statistically significant differences, others, such as the SF depth and concavity angle, did not consistently reach statistical significance. Thus, the clinical implications should be interpreted with caution. CBCT remains a valuable tool in preoperative evaluation, assisting clinicians in anticipating anatomical variability and minimizing surgical risks. Future research incorporating volumetric analysis and broader population samples is recommended to validate and expand upon these findings.

## Data Availability

No datasets were generated or analysed during the current study.
